# Repeated Daily Use of Dual-Light Antibacterial Photodynamic Therapy in Periodontal Disease—A Case Report

**DOI:** 10.3390/dj10090163

**Published:** 2022-09-01

**Authors:** Katherina Trujiilo, Ismo T. Räisänen, Timo Sorsa, Tommi Pätilä

**Affiliations:** 1 Department of Oral Diseases, Karolinska Institutet, 14152 Huddinge, Sweden; 2 Happident Mariberg, Gjörwellsgatan 26, 11260 Stockholm, Sweden; 3 Department of Oral and Maxillofacial Diseases, Faculty of Medicine, University of Helsinki and Helsinki University Hospital, 00290 Helsinki, Finland; 4 Department of Congenital Heart Surgery and Organ Transplantation, New Children’s Hospital, University of Helsinki, 00290 Helsinki, Finland

**Keywords:** antibacterial photodynamic therapy, oral hygiene, periodontitis

## Abstract

Good oral hygiene at home is the foundation for optimal treatment response and long-term periodontal disease control. Antibacterial photodynamic therapy (aPDT) provides a very potent adjunctive treatment for plaque control. However, the literature regarding repeated aPDT use is sparse. aPDT has been a modality applied mainly in the dental office environment, and when applied once a year or every few months, the results have been usually disappointing. Recently, LED development has brought aPDT for repeated and practical use at home. We present the very positive results and clinical outcome of daily applied dual-light aPDT-technology treatment in conjunction with mechanical cleaning of a 78-year-old male patient with severe periodontal disease (Stage IV and Grade B).

## 1. Introduction

The primary goal of the periodontal treatment is the elimination of pathogens on the nonshredding surfaces and to reduce and arrest the related inflammatory response and tissue destruction [1]. Thus, the traditional treatment of periodontitis in the clinic involves the anti-infective mechanical removal of dental plaque and mineralized deposits of calculus by scaling and root planing (SRP) from the supragingival and subgingival surfaces of the teeth without traumatizing these delicate surfaces [1,2]. Ultimately, the anti-infective periodontal treatment aims at disrupting and removing the dysbiotic biofilm structure and composition on the tooth/root surface to induce healing [2]. However, it should be noted that at all steps during the treatment, motivation, and adherence to careful oral hygiene at home form another cornerstone of effective long-term treatment results [1]. Effective self-care has been based on daily mechanical cleaning of the teeth, interdental spaces, and prosthetic structures of the pathogenic biofilm [1].

Antibacterial photodynamic therapy (aPDT) and antibacterial blue light (aBL) have emerged as solutions for attacking dental biofilm [1,3]. These methods are based on the absorption of photons of light in a specific light enhancer molecule, leading to electron transfer reactions that ultimately produce a bactericidal effect by reactive oxygen species [3]. While aPDT uses an externally brought molecule, aBL uses the exact mechanism with light enhancer molecules found naturally within the bacteria, primarily porphyrins and flavins [3,4,5]. The combination of aBL and aPDT has been shown to significantly increase the bactericidal action, especially on matured biofilms, and provide a sustained effect during repetitive application [3,4,5].

Traditionally, aPDT has been provided with laser devices in dental clinics. The use of laser-based aPDT is time-consuming and requires a steep learning curve. Most clinical study protocols have used a single or two treatments. Unsurprisingly, meta-analyses of the aPDT treatment applied in dental clinics have not shown improvement compared to traditional scaling and root planning or anti-infective treatment [1]. However, when the aPDT has been provided with indocyanine green (ICG)-based protocols, the results have been better. Most importantly, when the aPDT treatment has been regularly repeated for long enough, the results have significantly improved [6,7].

**Figure 1:**
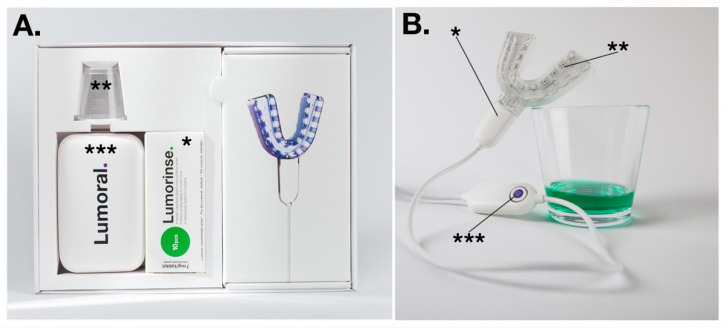
The dual-light aPDT device. (**A**) The packaging includes the effervescent ICG tablets (*) to be dissolved in 30 mL of water, for which a measuring cup (**) is available. A power source (***) provides electricity for the mouthguard-type light applicator. (**B**) The mouthpiece (*) is composed of 48 LED components (**) able to provide simultaneous 405 nm and 810 nm light. Symmetrically assembled LEDs provided light for both maxillary and mandibular dental arch. The button on the control unit provided a treatment time of 10 min (***). The glass contains dissolved mouth rinse.

Lumoral is an aPDT device designed for regular use at home. The device has an ICG mouthrinse, which is rinsed for one minute. The ICG attaches to the residual dental plaque [8]. After the rinsing, a light applicator activates the antibacterial effect. The light applicator provides a 50:50 combination of 405 nm aBL and 810 nm aPDT light. Please see Figure 1.

The aim of this case study was to investigate the results and clinical outcome of daily applied dual-light aPDT-technology treatment in conjunction with mechanical cleaning of a patient with severe periodontal disease.

## 2. The Patient Case

The patient is a 78-year-old male suffering from vascular dementia. In addition, the patient has hypertension, allergy, and epilepsy. He has suffered several cerebral hemorrhages since 2014, and he has two artificial heart valves and a permanent pacemaker implanted. He is a nonsmoker. His blood sugar has been at normal levels. He needs help with daily routines, and he performs tooth cleaning routines daily with the assistance of his wife. He has problems with dexterity, especially with his hands. He needs his wife’s help with toothbrushing, which is regularly performed twice daily with a manual toothbrush and regular toothpaste. He does not have any history of aPDT use before this study. During this study the patient had four visits where the anti-infective treatment with scaling and root planing was performed with manual curettes and ultrasonic cleaner/scaler, after which teeth were polished with prophylactic paste. Root planing with manual curettes was performed right after ultrasonic scaling in all periodontal pockets.

**Figure 2:**
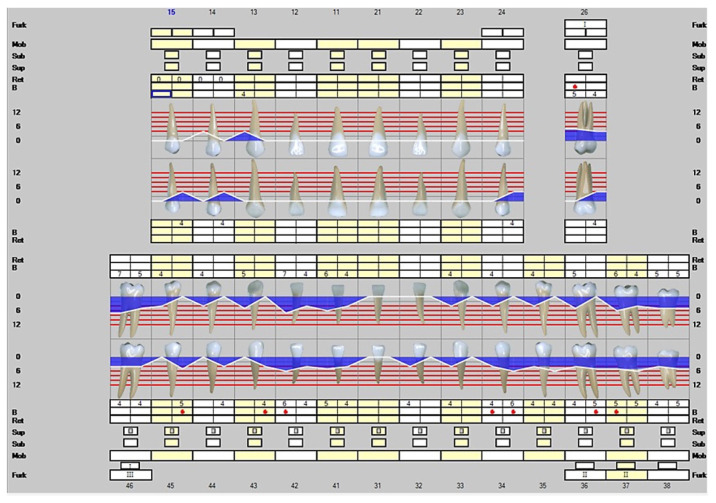
Representation of the clinical situation on 26 April 2021. The white line describes the clinical attachment level and the blue color infected periodontal pockets. The red dots represent a positive result in bleeding on probing.

The first visit to the dentist was on 26 April 2021. The patient’s periodontal status was unstable and had a lot of soft plaque. His periodontitis was at Stage IV and Gradus B, with seven teeth lost due to periodontitis. The radiographic bone loss showed Class II furcation involvement with extension up to the middle third of the maximally involved roots [9]. The disease was generalized and the periodontal tissue destruction commensurated with the biofilm deposits. There were a total of 43 moderately deep 4–6 mm periodontal pockets. The deepest probing depths were measured up to 7 mm and there were a total of two deep periodontal pockets. Please see Figure 2.

The dentist prescribed a dental hygienist visit on 1 October 2021, and scaling and root planing were performed. According to the hygienist assessment, his problem is everyday cleaning, and he develops a lot of soft plaque quickly, and food residuals are visible.

The second visit to the dentist was on 22 December 2021. Again, the periodontal status remained unstable. At this point, by the dentist’s recommendation, Lumoral dual-light aPDT treatment was started to support the oral hygiene regimen at home. Mechanical cleaning was recommended to be performed twice daily.

**Figure 3:**
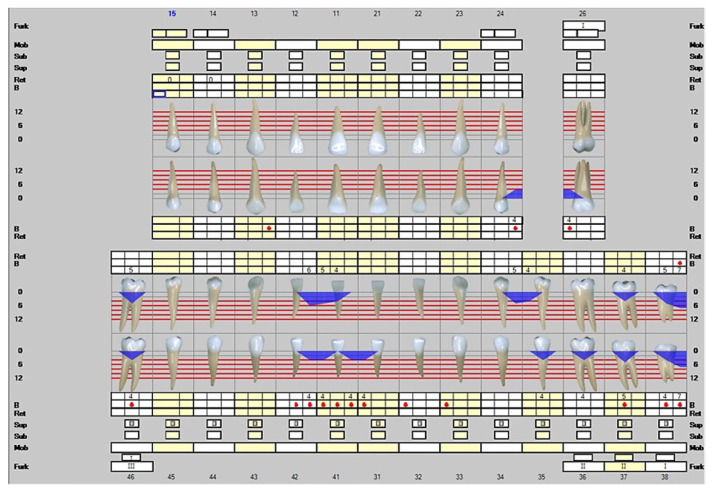
Representation of the clinical situation on 27 January 2022. The white line describes the clinical attachment level and the blue color plaque-infested periodontal pockets. The red dots represent a positive result in bleeding on probing. A total of eight teeth with a minimum of 4 mm pocket depths showed no pockets at all. In addition, the overall clinical attachment level improved significantly.

The third visit to the dentist was on 27 January 2022. At this point, the patient had used Lumoral daily for five weeks. Again, the scaling and root planing were repeated. Please see Figure 3. The number of moderately deep 4–6 mm periodontal pockets was 19. Two 7 mm deep periodontal pockets were found.

**Figure 4:**
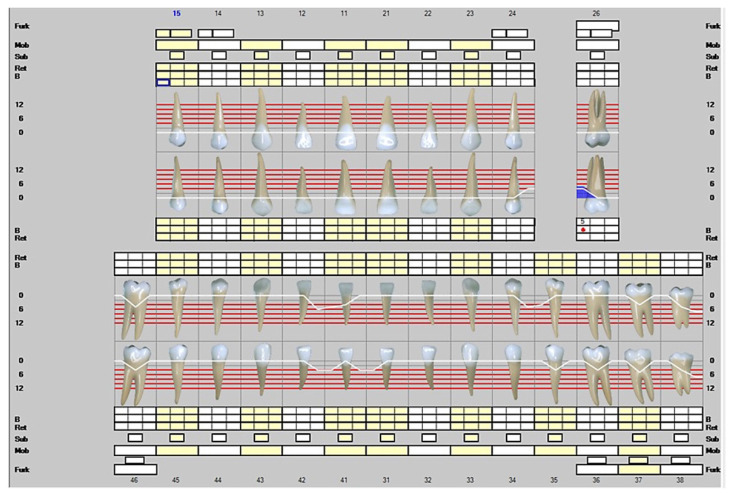
Representation of the clinical situation on 25 May 2022. The white line describes the clinical attachment level and the blue color infected periodontal pockets. The red dots represent a positive result in bleeding on probing.

**Figure 5:**
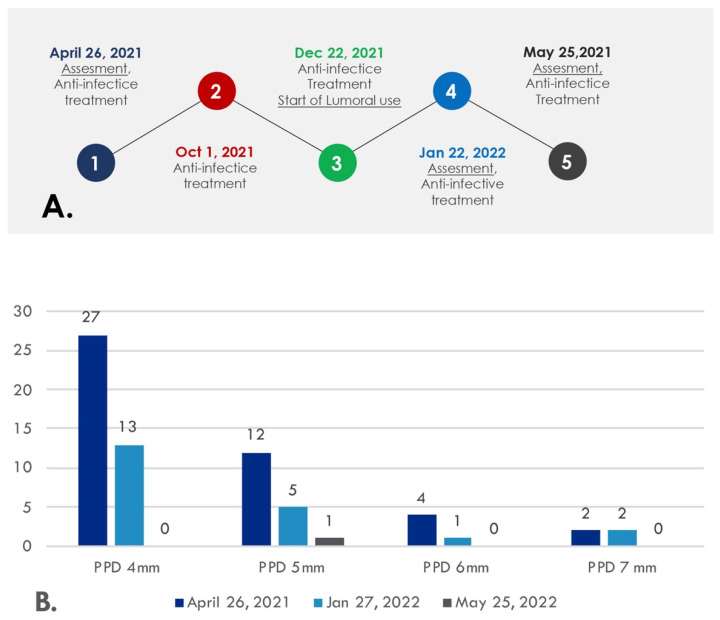
(**A**) The timeline of the patient visits to the clinic. (**B**) The number of periodontal pocket depths (PPD)s of 4 mm, 5 mm, 6 mm, and 7 mm measured at the beginning of the study (26 April 2021) and during aPDT use at the maintenance visits (27 January 2022 and 25 May 2022).

The fourth visit to the dentist was on 25 May 2022, after five months of Lumoral use. At this point, the patient had only a single infected pocket left, and the clinical attachment level was significantly improved. The infected periodontal pocket around the tooth 26 left was 5 mm deep. The teeth 27 and 28 were missing, while the opposing molars 37 and 38 were reserved. Please see Figure 4. Again, the scaling and root planing were repeated. The device use was easy, according to the patient and his custodian. Neither mild dementia nor the motoric disability after several strokes affected the use of the device under his wife’s guidance. Please see Figure 5 for a timeline for the patient visits to the clinic (A) and a summary of the beneficial changes and reductions recorded in periodontal probing depths during aPDT (B).

## 3. Discussion

This case report describes a periodontitis patient with difficulties maintaining mechanical oral hygiene. The patient received continuous aPDT treatment for 6–7 months, using the device every other day. The ability of the patient to keep his periodontal pockets infection-free indeed improved and enhanced after the device use. The number of deep periodontal pockets reduced from two 7 mm pockets to zero, and the number of moderately deep periodontal pockets reduced from 43 to 1. At the same time, the clinical attachment level improved significantly during the treatment.

The most important difference to the existing aPDT treatments in Lumoral use is the consistency of the treatment application. In most publications to date, the aPDT is reported to be given only once. Lumoral enables continuous use. In the study by Giannelli et al. [6], the in-office aPDT treatment was repeated weekly up to 10 times. In the split quadrant protocol, after the initial phase, the separately applied aPDTs included 655 nm light with Toluidine blue with additionally applied 405 nm aBL. At one year, the number of >7 mm deep pockets was reduced from a mean of 15.0 to 0.6 in the treatment quadrants, while in the sham quadrants, it was reduced from 12.9 to 5.7. The reduction in the number of moderately deep pockets, 4–6 mm, in the treatment quadrants reduced from a mean of 32.7 to 2.0, while in the sham treated quadrants, the moderately deep pockets increased from 28.5 to 36.1 at one year [6]. In the later phase, at four years, the clinical attachment and pocket depths were significantly better in the treated compared to the sham treated quadrants. The effect at four years was greater than the observed effect at one-year follow-up [7].

In most cases, nonsurgical periodontal treatment brings significant clinical improvements. However, complete removal of bacterial deposits has been and still is very challenging to achieve, especially if the disease has formed deep periodontal pockets [1]. Eventually, the remaining bacterial biofilm may disturb the healing of periodontal tissues by upregulating the inflammation [1]. Treatment of severe periodontitis may require antimicrobial or surgical treatment in addition to mechanical scaling and root planing anti-infective therapy [1].

Systematic reviews have demonstrated the clear benefits of antiseptics in managing gingival diseases. In a recent meta-analysis including 70 studies, adjunctive antiseptics in mouthrinses and dentifrices reduced gingival indices, bleeding on probing, and the amount of plaque [10]. However, many of the products available have inherent drawbacks. Using a mouthrinse that affects the whole mouth can disturb the oral microbiome. On the other hand, chlorhexidine stains the teeth and can disturb taste senses. The use of antibiotics is restricted to the development of antimicrobial resistance formation [3]. Oral irrigation has been available for decades as an adjunctive home therapy for oral hygiene. In meta-analysis it has shown a trend toward improved gingival health over regular oral hygiene or toothbrushing only, despite not reducing dental plaque [11]. The aPDT exerts a very potential antimicrobial efficacy without resistance formation. It has also been safe during tens of years of use in clinical dentistry.

The Lumoral device is able to provide aPDT for supragingival plaque, but also through the periodontal tissue into the periodontal pocket, a method called transgingival light application. The Lumoral Clinic device is a low-profile mouthpiece in which 48 high-power LEDs are enclosed. Each LED unit provides 405 nm and 810 nm infrared light. The 405 nm blue light hardly penetrates tissue, but the 810 nm near-infrared light penetrate significantly better [12]. The depth of the penetration of ICG mouth rinse in the dental pockets would have an effect of the antibacterial action within the pockets.

The product is used in combination with an oral rinse, Lumorinse, which is packaged in tablet form. The Lumorinse tablet includes 7 mg of ICG, which is mixed with water in a 30 mL measuring cup to provide a solution. The used photosensitizer, ICG, is a tri-carboxy-cyanine that belongs to a large family of cyanine dyes with a peak absorption rate of 805–810 nm wavelength. ICG has both hydrophilic and lipophilic properties. It is an FDA-approved photosensitizer for medical diagnostics, and it has been used for oncologic photodynamic therapy and photothermal therapy [13]. Recently, ICG has gained special attention in dentistry for its antibacterial properties. The mechanism of action of ICG is different from other photosensitizers. ICG has been described as a low producer of reactive oxygen. The light-activated ICG releases roughly 85% of the absorbed energy as heat, and 15% of the energy is transferred as reactive oxygen species if oxygen is available. The thermal effect is considered to be part of the antibacterial act, described as photothermal antibacterial action [13].

Bashir et al. performed a systematic review evaluating the efficacy of ICG-based aPDT as an adjunctive treatment to the conventional scaling and root planing (SRP) in periodontal patients, when compared to the nonsurgical periodontal treatment alone. They reported a mean additional pocket depth reduction of 1.17 mm at 3 months and a mean additional reduction of 1.06 mm 6 months, when compared to the SRP alone. For CAL, the adjunctive ICG-PDT resulted in a mean additional gain of 0.70 mm at 3 months and a mean additional gain of 1.03 mm at 6 months [14].

The home-use dual-light aPDT device was feasible and handy when used by a disabled person who could not take complete care of his oral hygiene. Neither mild dementia nor the motoric disability after several strokes affected the use of the device under his wife’s guidance. The patient and his caregiving wife felt relief from the results achieved. This indeed encourages the use of such a device in other disabled populations. Such populations include elderly care patients, where oral hygiene has shown to be disappointing in several studies [15]. In addition, the nursing staff commonly regards performing oral hygiene treatment as a challenging and unpleasant task [15]. Thus, Lumoral devices could conveniently help to improve oral health in elderly homes.

The recovery from an unstable Stage IV periodontal disease during the 13-month surveillance period was excellent. There was a clear improvement in oral hygiene and a significant reduction in infected pockets. In addition, the clinical attachment level improved. There was only one residual infected periodontal pocket left, and the etiology of this residual pocket can be argued to be at least partly due to occlusion trauma. In that regard, the authors recognize that patient level and site-specific active matrix metalloproteinase (aMMP)-8 oral fluid analysis could have provided beneficial information not only about the periodontal inflammation status in that site but also as a whole. Previous studies have shown a significant association between site-specific aMMP-8 levels and the diagnosis of periodontitis and prediction of treatment outcome [16,17]. Moreover, a significant, positive association between mouthrinse aMMP-8 levels and the stage and grade of periodontitis and a positive treatment effect [18,19] have been shown. Thus, monitoring aMMP-8 levels in both mouthrinse and gingival crevicular fluid during periodontal treatment phase provides information to assess treatment response and maintenance therapy, as the more the aMMP-8 levels are elevated, the more severe the disease [16]. As this is a case study, generalization of the current results to the wider population is not possible. Nevertheless, the excellent results from this case report study support conducting further studies in different, larger populations with various cases of periodontal conditions to confirm the current results and to extend our knowledge on the relationship between aPDT, patient level, and site-specific aMMP-8 levels, and periodontal treatment effect.

## 4. Conclusions

Repeated aPDT treatment at home can improve oral hygiene and the results of periodontal treatment. Further studies are required to confirm the current results.

## Data Availability

Not applicable.
